# Effectiveness of inbuilt cell phone reminders in chronic medication compliance

**DOI:** 10.4102/safp.v67i1.6031

**Published:** 2025-04-29

**Authors:** Thanduxolo P. Myeni, Somasundram Pillay

**Affiliations:** 1 Department of Internal Medicine, Faculty of Health Sciences, University of KwaZulu-Natal, Durban, South Africa

**Keywords:** cell phone reminders, medication adherence, chronic disease management, compliance, digital health interventions

## Abstract

**Background:**

Adherence to chronic medication is crucial for managing chronic diseases and preventing complications. However, maintaining consistent adherence remains challenging, particularly in low- and middle-income countries where forgetfulness is a common barrier. The widespread use of mobile phones, even in resource-limited settings, offers a practical opportunity to leverage inbuilt reminder features to support medication adherence. This study evaluates the effectiveness of inbuilt cell phone reminders in enhancing chronic medication compliance among patients in the eThekwini health district of KwaZulu-Natal, South Africa.

**Methods:**

In this prospective, cross-sectional study, 400 patients on chronic medications were systematically selected from five healthcare centres. Participants were divided into two groups: those using mobile phone reminders (Group 1) and those who did not (Group 2). Medication adherence was assessed using a standardised questionnaire, and statistical analyses, including Chi-square tests and logistic regression, were conducted to identify differences in adherence rates.

**Results:**

Group 1 exhibited significantly higher adherence rates (87%) compared to Group 2 (67%, *p* < 0.001). The use of cell phone reminders was associated with a 2.5-fold increase in the odds of adherence (odds ratio [OR] = 2.5, 95% confidence interval [CI]: 1.7–3.6, *p* < 0.001).

**Conclusion:**

Inbuilt cell phone reminders are a cost-effective intervention that significantly enhances medication adherence, especially in resource-limited settings. Integrating mobile technologies into public health strategies could improve chronic disease management.

**Contribution:**

This study highlights the potential of mobile phone reminders as a practical tool for improving medication adherence, with significant implications for public health strategies in low-resource settings.

## Introduction

Chronic non-communicable diseases (NCDs) refer to a group of slowly progressive medical conditions or diseases that are long in duration (chronic) and are non-infectious and non-transmissible among people (non-communicable). Non-communicable diseases are the leading cause of mortality worldwide, accounting for approximately two-thirds of all deaths globally.^1^ According to the World Health Organization (WHO), each year, more than 15 million people between the ages of 30 years and 69 years die from NCDs, with 85% of these ‘premature’ deaths occurring in low- and middle-income countries.^2^ In 2015, commitments related to NCDs, such as promoting physical activity, mental health, well-being and achieving universal health coverage, were incorporated into the 2030 Agenda for Sustainable Development.^3^

To effectively manage chronic conditions and prevent disease progression and complications, it is crucial that prescribed medication is taken regularly to maintain therapeutic drug levels in the body. Inconsistent medication intake can lead to suboptimal drug levels, reducing the effectiveness of treatment. Medication compliance is defined as the degree or extent of conformity to the recommendations regarding day-to-day treatment, including timing, dosage and frequency.^4^ It is also described as ‘the extent to which a patient acts in accordance with the prescribed interval and dose of a dosing regimen’.^4^ Compliance has been further defined as ‘the extent to which a person’s behaviour coincides with medical advice’.^5^ Adherence and compliance is often used interchangeably in medical literature. Non-compliance, therefore, essentially means that patients do not follow the advice of their healthcare providers.^6^ The success of any therapeutic regimen depends on the compliance of the individual involved. The efforts of healthcare providers can only yield the desired effects if patients adhere to their medication regimen. Unfortunately, medication non-compliance, with its associated detrimental effects, is becoming increasingly prevalent, especially among patients with chronic diseases.^7^

Numerous factors contribute to poor medication adherence, including patient-related factors (e.g., suboptimal health literacy and lack of involvement in the treatment decision-making process), physician-related factors (e.g., prescription of complex drug regimens, communication barriers, ineffective communication of information about adverse effects and care provided by multiple physicians) and healthcare system-related factors (e.g., limited time for office visits, restricted access to care and lack of health information technology).^8^

Poor adherence to long-term therapies severely compromises treatment effectiveness, making it a critical concern in population health, affecting both qualities of life and health economics. Interventions to improve adherence could yield a significant positive return on investment through primary prevention (of risk factors) and secondary prevention of adverse health outcomes. The consequences of non-adherence include worsening conditions, increased comorbid diseases, higher healthcare costs and increased mortality. Addressing non-adherence requires collaboration between healthcare practitioners and patients to enhance adherence and achieve optimal health outcomes.^9^ Healthcare professionals, including physicians, pharmacists and nurses, play a significant role in their daily practice in improving patient medication adherence.^10^ Ensuring appropriate medication adherence helps to prevent severe relapses, antibiotic resistance and avoidable hospitalisations.^11^

Multiple studies have explored the use of mobile devices in healthcare, leading to the development of various technological approaches aimed at expanding health encounters and improving health promotion across a range of medical conditions. In South Africa, where landlines are scarce, and the population is highly mobile, cellular phones are widely used. The use of cellular phones in human immunodeficiency viruses (HIV) and/or acquired immunodeficiency syndrome (AIDS)-related healthcare has received particular attention, with telecommunication strategies in resource-limited settings becoming a key focus on the international public health agenda.^12^

However, there is limited research specifically focussing on South Africa. In 2007, Crankshaw et al. conducted a study titled ‘Exploring the patterns of use and the feasibility of using cellular phones for clinic appointment reminders and adherence messages in an Antiretroviral treatment clinic in Durban, South Africa’, which found that ‘most respondents were willing for the clinic to contact them on their cell phones either verbally (99%) or via text messages (96%)’.^12^ No other research specifically addressing the effectiveness of cell phone reminders in improving chronic medication compliance in South Africa was identified online.

Given the scarcity of local data, this study aims to establish whether there is a relationship between the use of inbuilt mobile device reminders and medication compliance and to measure the effectiveness of these reminders in promoting adherence to chronic medication. A reminder, by definition, is ‘something (such as a note or notification) designed to prompt or aid the memory’^13^ and in the context of this study, it is a cell phone reminder programmed by the user to notify them when its time to take their medication.

## Research methods and design

The study utilised a prospective, cross-sectional, observational quantitative design to investigate the relationship between the use of inbuilt cell phone reminders and chronic medication compliance among patients in the eThekwini health district of KwaZulu-Natal, South Africa. Creswell^14^ emphasises that a quantitative approach is appropriate when the research goal is to understand relationships between variables. Given that this study aimed to explore the relationship between cell phone reminders and medication compliance using questionnaires, an observational quantitative approach was deemed the most suitable.

The study sample was drawn from a population of willing participants from five selected healthcare centres within the eThekwini health district. Participants who met the inclusion criteria – being over the age of 18 years, on chronic medication, and presenting to outpatient departments during the study period – were eligible for inclusion.

Healthcare centres within the eThekwini health district were categorised according to the level of care they provide: district, secondary and tertiary care. Systematic cluster random sampling was used to select one healthcare centre from each level of care to ensure a diverse and representative sample. The study included five healthcare centres: a local clinic, a community health clinic (CHC), a district hospital, a regional hospital and a tertiary hospital, namely Svananda Clinic, KwaMashu CHC, Wentworth Hospital, RK Khan Hospital and King Edward Hospital. Non-probability convenience sampling was employed to select the first 80 willing participants from each healthcare centre, resulting in a total study size of 400 participants. These participants were asked to complete a structured questionnaire designed to assess their medication adherence and the use of cell phone reminders.

### Data collection and statistical analysis

Participants were divided into two groups: those who used cell phone reminders and those who did not. Medication compliance in these two groups was assessed using a questionnaire and with the help of a health professional. The primary data collection tool was a simplified questionnaire available in both English and isiZulu, with the isiZulu version translated by a professional language practitioner to maintain validity and accuracy. This self-report method was both cost-effective and practical for use in outpatient settings.

The collected data were analysed using descriptive statistics to summarise demographic information and adherence behaviours. Inferential statistics, including Chi-square tests for categorical data and *t*-tests or Wilcoxon rank-sum tests for numeric data were employed to determine the statistical significance of differences between the groups. All statistical analyses were performed using Statistical Package for Social Sciences (SPSS) version 29, with a *p*-value of less than 0.05 considered statistically significant. This rigorous methodological approach aimed to yield reliable insights into the effectiveness of mobile phone reminders in enhancing medication adherence among patients with chronic diseases.

### Ethical considerations

Ethical approval for this study was obtained from the Biomedical Research Ethics Committee (BREC) (Reference number: BREC/00005063/2022) of the University of KwaZulu-Natal. Further ethics approval was secured from the KwaZulu-Natal Department of Health through the Provincial Health Research and Ethics Committee (PHREC). The eThekwini Health District also granted ethical approval to conduct research within the district. Individual institutions provided either verbal or written approval.

Participants were provided with an information sheet and consent form that explained the purpose of the study. Participation was entirely voluntary, and the confidentiality of the information provided was assured. The questionnaires were completed anonymously, and participants were informed that their responses would not affect their future treatment at the facility. The data collected were to be used strictly for research purposes to aid in health promotion. Written informed consent was obtained from all the participants of the study. Contact details for the principal investigator and the university were provided on the information sheet. All participants were over 18 years of age and recruitment was based on their willingness to participate, provided they were on chronic medication.

## Results

### Demographics

This section of the questionnaire sought to gather data on the demographics of the research. The results are displayed in Table 1. The gender distribution revealed a higher proportion of female patients, comprising 62.3% (*n* = 249) of the sample, compared to 37.8% (*n* = 151) of male patients (*p* < 0.001). This trend indicates a notable gender disparity in the study sample. Age distribution showed that the majority of patients were in the 50–59 years age group, accounting for 29% (*n* = 116), followed closely by the 60–69 years age group at 27% (*n* = 108) and the 40–49 years age group at 22.8% (*n* = 91). The younger age groups, particularly those under 30 years, had significantly fewer patients, with 3.5% (*n* = 14) for ages 20–29 years and 0.5% (*n* = 2) for those under 20 years (*p* < 0.001).

**TABLE 1:** Demographics of study population.

Demographic variable	*n*	%	*p*
**Gender**	-	-	< 0.001
Female	249	62.3	-
Male	151	37.8	-
**Age category (years)**	-	-	< 0.001
< 20	2	0.5	-
20–29	14	3.5	-
30–39	37	9.3	-
40–49	91	22.8	-
50–59	116	29.0	-
60–69	108	27.0	-
70–79	28	7.0	-
80–89	4	1.0	-
**Education**	-	-	< 0.001
No schooling	21	5.3	-
Primary school	75	18.8	-
High school	269	67.3	-
Tertiary	35	8.8	-
**Residence**	-	-	< 0.001
Inanda	82	20.5	-
KwaMashu (A, B, C, D, E, F)	59	14.8	-
Chatsworth	51	12.8	-
Wentworth	49	12.3	-
Bluff	29	7.3	-
Ntuzuma	23	5.8	-
Lamontville	15	3.8	-
Shallcross	15	3.8	-
Jacobs	12	3.0	-
Mayville	12	3.0	-
Montclair	11	2.8	-
Clairmont	8	2.0	-
Savanna Park	7	1.8	-
Marianhill	7	1.8	-
uMbilo	4	1.0	-
Bothas Hill	4	1.0	-
Hillary	4	1.0	-
Clairwood	4	1.0	-
Newlands	4	1.0	-

Educational attainment among patients demonstrated that the majority had completed high school, comprising 67.3% (*n* = 269), while a smaller proportion had attained tertiary education at 8.8% (*n* = 35) and an even smaller proportion had no schooling at 5.3% (*n* = 21). Primary school education was reported by 18.8% (*n* = 75) of patients (*p* < 0.001).

Regarding residence, the patients were widely distributed across various areas, with the highest proportions residing in Inanda at 20.5% (*n* = 82), KwaMashu at 14.8% (*n* = 59) and Chatsworth at 12.8% (*n* = 51). Other areas, such as Ntuzuma at 5.8% (*n* = 23) and Lamontville at 3.8% (*n* = 15), had fewer patients (*p* < 0.001).

### Chronic medication

**FIGURE 1:**
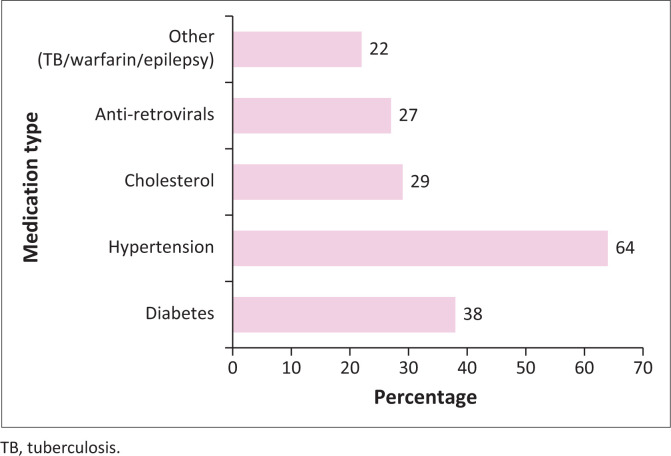
Chronic medications.

The majority of patients involved in the study were predominantly on medication for hypertension and diabetes (Figure 1). These conditions were the most commonly managed chronic diseases among the participants. A smaller proportion of patients were on other medications, including those for the management of conditions requiring warfarin therapy for anticoagulation, anti-epileptics and tuberculosis (TB) treatment. This distribution reflects the prevalent chronic health issues within the study population and highlights the need for adherence to these critical long-term therapies.

### Cell phone reminder use

**FIGURE 2:**
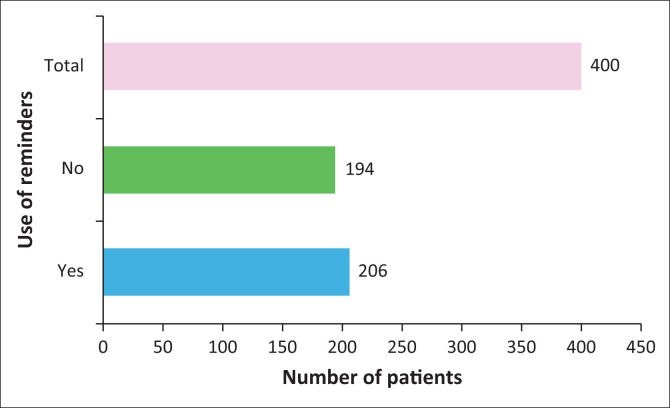
Cell phone reminder use.

A total number of 400 patients were involved in this study. Patients were asked if they used cell phone reminders or not. A total of 206 participants responded ‘YES’ to the question and were labelled as ‘Group 1’ participants. The other participants who answered ‘NO’ to the question were labelled as ‘Group 2’ participants. The data revealed a nearly equal distribution between individuals who use a cell phone reminder to manage their medication adherence and those who do not, with no statistically significant difference between the groups (*p* = 0.549). Specifically, 51.5% of the patients (*n* = 206) reported using a cell phone reminder, while 48.5% (*n* = 194) did not use such reminders. This close distribution provides a balanced comparison between the two groups, facilitating an effective analysis of the impact of cell phone reminders on medication compliance (Figure 2).

### Non-adherence

Both groups of participants were asked a series of questions designed to assess their adherence behaviours. The questions aimed to explore common factors that may influence medication adherence. The specific questions posed were:

- *Question 1: Do you ever forget to take your medication?*
- *Question 2: Are you careless at times about taking your medication?*
- *Question 3: Sometimes if you feel better, do you stop taking your medication?*

**FIGURE 3:**
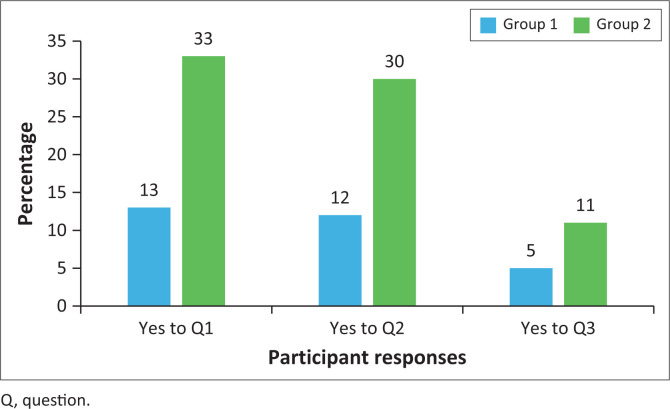
Comparison of responses for Group 1 and Group 2.

The analysis of adherence behaviours among the study participants revealed significant differences between the two groups (Figure 3):

- *Forgetfulness*: When asked, ‘Do you ever forget to take your medication?’, 33% of participants from Group 2 answered ‘Yes’. In contrast, only 13% of participants from Group 1 reported forgetting to take their medication. This difference was statistically significant (*p* < 0.001), indicating that cell phone reminders may effectively reduce forgetfulness in medication adherence.
- *Carelessness*: In response to the question, ‘Are you careless at times about taking your medication?’, 30% of Group 2 participants admitted to being careless, compared to only 12% of Group 1 participants. This difference was also statistically significant (*p* < 0.001), suggesting that cell phone reminders may help mitigate carelessness in taking medication.
- *Discontinuation when feeling better*: Finally, when asked, ‘Sometimes if you feel better, do you stop taking your medication?’, 11% of Group 2 participants responded ‘Yes’, whereas only 5% of Group 1 participants reported discontinuing their medication under such circumstances. This difference was again statistically significant (*p* < 0.001), highlighting the potential role of cell phone reminders in preventing premature discontinuation of medication (Figure 3).

### Recent medication non-adherence

The concluding section of the questionnaire was designed to assess recent instances of non-adherence as self-reported by the participants. The following three questions were posed to elicit this information:

- *Question 4: Did you not take any of your medicine over the past weekend?*
- *Question 5: Thinking about the last week. How often have you not taken your medicine?*
- *Question 6: Over the past 3 months, how many days have you not taken any medicine at all?*

When participants in Group 1 were asked, ‘Did you not take any of your medicine over the past weekend?’, 10% responded affirmatively (‘Yes’), while a similar proportion of 12% from Group 2 also reported non-adherence.

In response to the question, ‘Thinking about the last week, how often have you not taken your medicine?’, 90% of Group 1 participants indicated that they had never missed their medication, in contrast to only 70% of Group 2 participants.

Furthermore, a substantial 26% of patients in Group 2 reported having missed their medication on at least three or more occasions over the past 3 months, compared to only 4% of patients in Group 1.

**FIGURE 4:**
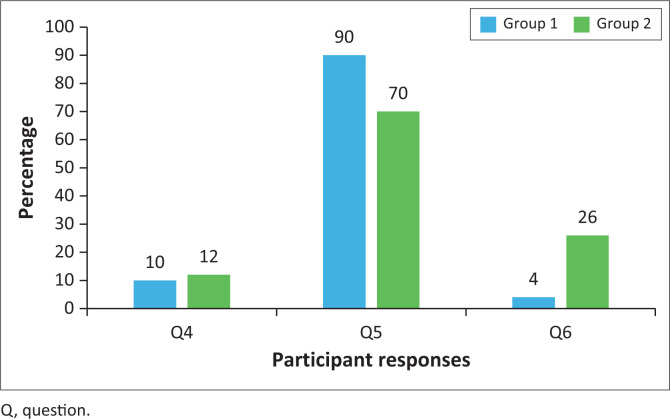
Comparison of Group 1 and Group 2 responses.

Collectively, these statistical findings indicate that Group 1 participants, who utilised cell phone reminders, demonstrated greater adherence to their medication regimen compared to Group 2 participants (Figure 4).

**FIGURE 5:**
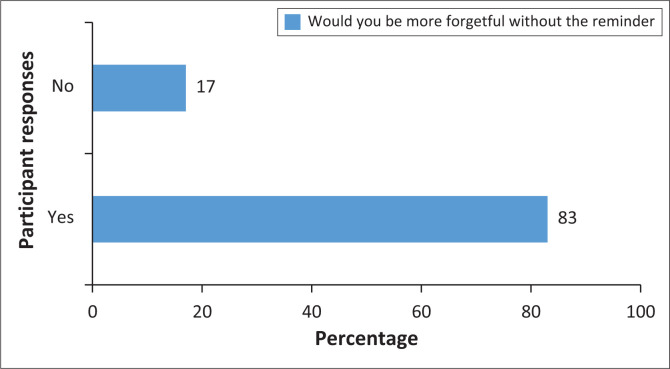
Forgetfulness without cell phone reminders (Group 1).

Group 1 participants were subsequently asked whether they believed they would be more forgetful in the absence of cell phone reminders (Figure 5). The data indicate a significant reliance on these reminders for medication adherence among Group 1 participants. Specifically, 83% of those using cell phone reminders believed they would experience increased forgetfulness without them, whereas only 17% felt they would not be more forgetful in their absence.

### Advanced statistical analysis

#### Analysis of medication adherence in relation to cell phone reminder use

Table 2 demonstrates that individuals who forget to take their medication are significantly less likely to use a cell phone reminder (*p* < 0.001) and the following noteworthy findings are also noted:

- Patients who are careless about taking their medication are significantly less likely to use a cell phone reminder, with the odds reduced by approximately 70% (*p* < 0.001).
- The unadjusted model also demonstrates that stopping medication when feeling better is associated with a lower likelihood of using a cell phone reminder, reducing the odds by about 58% (*p* = 0.029).
- There was no significant association between not taking medication over the past weekend and the use of a cell phone reminder (*p* = 0.489).
- Those who had many days of non-adherence in the past 3 months were significantly less likely to use a cell phone reminder, with the odds reduced by 89% (*p* < 0.001).

**TABLE 2:** Unadjusted model results.

Variable	Chi-square	Chi-Square (*p*)	Unadjusted			
			Exp(B)	95% CI for Exp(B)		Sig.
				Lower	Upper	
Q3: Do you ever forget to take your medication?	22.763	< 0.001	0.300	0.180	0.500	< 0.001
Q4: Are you careless at times about taking your medication?	21.387	< 0.001	0.302	0.179	0.510	< 0.001
Q5: Sometimes if you feel better, do you stop taking your medication?	4.981	0.038	0.420	0.193	0.917	0.029
Q6: Did you not take any of your medication over the past weekend?	0.480	0.521	0.799	0.424	1.507	0.489
Q7: Over the past 3 months, how many days have you not taken any medication at all?	39.886	< 0.001	0.113	0.052	0.246	< 0.001
Q8: Thinking about the last week, how often have you not taken your medication?	30.320	< 0.001	-	-	-	< 0.001
1–2 times	-	-	0.319	0.175	0.579	< 0.001
3–5 times	-	-	0.048	0.006	0.371	0.004
6–10 times	-	-	0.363	0.033	4.043	0.410

Exp(B), exponentiated beta; CI, confidence interval; Sig., significance; Q, question.

Table 3 shows that after adjusting for confounders, the odds of using a cell phone reminder are still significantly lower (about 70% reduction) for those who forget their medication (*p* < 0.001). In addition, other important findings include:

- Carelessness about taking medication continues to be significantly associated with lower odds (about 70%) of using a cell phone reminder (*p* < 0.001).
- Additionally, Table 3 demonstrates that the significant association persists, with a 56% reduction in the odds of using a cell phone reminder when people stop taking medication because they feel better (*p* = 0.044).
- Moreover, no significant association was found between weekend non-adherence and the use of a cell phone reminder (*p* = 0.552).
- Notably, the strong association remains, with a significant reduction (about 89%) in the odds of using a cell phone reminder for those with many days of non-adherence over the past 3 months (*p* < 0.001).

**TABLE 3:** Adjusted model results (adjusted for gender, age and education).

Variable	Chi-square	Chi-Square (*p)*	Adjusted			
			Exp(B)	95% CI for Exp(B)		Sig.
				Lower	Upper	
Q3: Do you ever forget to take your medication?	22.763	< 0.001	0.296	0.175	0.499	< 0.001
Q4: Are you careless at times about taking your medication?	21.387	< 0.001	0.303	0.176	0.521	< 0.001
Q5: Sometimes if you feel better, do you stop taking your medication?	4.981	0.038	0.439	0.197	0.978	0.044
Q6: Did you not take any of your medication over the past weekend?	0.480	0.521	0.824	0.435	1.560	0.552
Q7: Over the past 3 months, how many days have you not taken any medication at all?	39.886	< 0.001	0.107	0.048	0.236	< 0.001
Q8: Thinking about the last week, how often have you not taken your medication?	30.320	< 0.001	-	-	-	< 0.001
1–2 times	-	-	0.279	0.148	0.527	< 0.001
3–5 times	-	-	0.032	0.004	0.257	0.001
6–10 times	-	-	0.189	0.015	2.353	0.195

Exp(B), exponentiated beta; CI, confidence interval; Sig., significance; Q, question.

These results suggest that individuals who exhibit certain non-adherent behaviours, such as forgetting, carelessness and stopping medication when feeling better, are less likely to use cell phone reminders. Alternatively, those who are more adherent, are more likely to use cell phone reminders.

## Discussion

Medication adherence is a multifaceted challenge that necessitates comprehensive strategies for improvement. Enhancing adherence to prescribed medications may exert a more significant impact on public health than the discovery of new therapies.^15^ Interventions designed to improve medication adherence, particularly in resource-constrained settings, are critically important and warrant thorough investigation. The widespread availability and usage of mobile phones in many low- and middle-income countries present a promising opportunity. As reported in the 2002 Stats South Africa General Household Survey, 88.7% of South African households rely exclusively on cellular phones.^16^ This widespread access to mobile technology can be strategically utilised by implementing built-in cell phone reminder tools or alarms, a feature available even in basic entry-level phones at no additional cost.

Globally, research into the use of mobile phone reminders for improving medication adherence has yielded promising results. Studies conducted in Malaysia and South India demonstrated that patients who received cell phone reminders showed improved adherence to their medications.^17,18^ A systematic electronic database search by Isaac Amankwaa et al.^19^ focussed on randomised controlled trials (RCTs) and quasi-experimental studies assessing the effectiveness of mobile phone-based interventions for antiretroviral therapy (ART) adherence. The analysis concluded that ‘scheduled mobile phone text messaging has demonstrated significant improvement in adherence to ART’.^19^ The findings of this study align closely with the results of these other key investigations.

Our data indicate that participants in Group 1, who utilised cell phone reminders, demonstrated superior medication compliance and exhibited lower rates of forgetfulness compared to those in Group 2. A higher percentage of patients in Group 2 admitted to occasionally forgetting to take their medication, with a significant difference in forgetfulness rates observed between the two groups. The reduced forgetfulness among Group 1 participants may be attributed to the use of reminders, effective personal routines or a strong commitment to their treatment plans. These findings align with current available global literature, where Rodrigues et al. similarly reported improved adherence among patients undergoing anti-retroviral treatment when cell phone reminders were employed.^18^ This key finding supports the role of cell phone reminders in enhancing medication compliance.

Moreover, the data reveal that a greater percentage of Group 2 participants acknowledged being occasionally careless about taking their medication. This notable subset of the population may benefit from additional support to enhance their adherence behaviours. In addition, Group 2 participants were more likely to have missed their medications on certain days, whereas a significantly lower percentage of Group 1 participants reported missed doses. This consistent medication-taking behaviour among Group 1 participants underscores strong adherence within this group, suggesting that effective management strategies, including the use of reminders, play a crucial role.

A significant proportion of patients recognised the utility of digital adherence aids in managing their medication schedules. Notably, 83% of Group 1 participants indicated they would be more forgetful without cell phone reminders, reflecting a strong perceived reliance on these digital aids (*p* < 0.001). This dependence underscores the critical role of cell phone reminders in enhancing medication adherence by mitigating forgetfulness. In contrast, the 17% of Group 1 participants who did not believe they would be more forgetful without reminders may employ alternative strategies or possess high intrinsic motivation and cognitive ability to adhere to their medication schedules. This minority group highlights that while digital aids are beneficial, they are not universally necessary for all individuals.

The utilisation of cell phone reminders represents a positive step towards improving medication adherence, as these digital aids provide consistent and timely prompts, reducing the likelihood of missed doses. A meta-analysis of randomised clinical trials assessing the effect of mobile telephone text messaging on medication adherence in chronic disease conducted by Thakkar Jay et al. found that ‘mobile phone text messaging approximately doubles the odds of medication adherence’.^20^

While most individuals maintain good adherence to their medication schedules, a significant portion of Group 2 participants were less compliant. This finding underscores the need to explore and address the barriers faced by those who do not use or benefit from such tools in future research, ensuring that all patients receive the necessary support for optimal medication adherence. These individuals may rely on other methods for medication adherence or face barriers to accessing or using digital tools. Addressing this issue could involve implementing more widespread use of digital reminders, educational programmes, targeted interventions or other adherence-enhancing strategies to improve health outcomes for all patients.

Overall, the impact of this technological intervention on medication adherence is highlighted by contrasting the findings between Group 1 and Group 2 participants. The higher rates of forgetfulness and non-compliance observed among Group 2 participants further emphasise the effectiveness of reminders in maintaining better compliance rates. The results from this study align with other global and local studies, supporting the consideration of this simple yet cost-effective technology as a scalable solution to a pervasive problem in chronic disease management.

### Study limitations

Despite the strengths of the study, including diversity, fair sample size and the high replicability of the chosen research methodology, several limitations are worth mentioning. Firstly, patients who feel the need to set cell phone reminders of their own volition are possibly more enthusiastic about their health and the adherence to medication in this group may inherently be better as compared to less health-conscious individuals. Secondly, while the use of self-reported questionnaires as a data collection method is cost-effective and practical for use in an outpatient setting, it is subject to bias, including recall bias and reporting bias, which may overestimate adherence.

The WHO commonly classifies adherence assessment methods into subjective and objective categories. Subjective methods involve the patient’s self-assessment of their medication-taking behaviour or healthcare provider assessments, typically via questionnaires. Objective methods, such as measurements of clinical outcomes, dose counts, pharmacy records and electronic monitoring of medication administration, are generally considered more reliable in measuring treatment adherence.^21^

Further classification distinguishes between direct and indirect methods of assessment. Direct methods involve the direct observation of therapy or the measurement of drug (or metabolite) levels or biological markers in blood or urine, which confirm medication intake by the patient. Indirect methods include patient questionnaires, self-reports, pill counts, prescription refill rates, assessment of clinical response, electronic medication monitors and measurement of physiologic markers or patient diaries.^20^ These indirect methods, such as electronic pill counts and electronic pill bottles may offer more accurate results than questionnaires. However, they are very costly to set up, labour-intensive and do not provide actual evidence that the pill is ingested. To obtain highly accurate results, future research may benefit from employing direct methods of assessing compliance such as monitoring drug levels or drug metabolites. While this may be more costly and invasive, this is the most accurate form of assessing adherence as it proves the ingestion of the drug. Furthermore, future research directed at cell phone reminder use and chronic medication compliance should contemplate a qualitative approach to explore and understand patients’ attitudes regarding the use of reminders as an aid.

## Conclusion

Given the high penetration of mobile phones even in resource-limited settings, leveraging inbuilt reminder features could provide a practical method to support patients in adhering to their prescribed medication regimens. A large percentage of participants recognise the utility of cell phone reminders as a free and relatively simple digital adherence aid in managing their medication schedules. They believe that these reminders play a crucial role in their medication adherence strategies, supporting the effectiveness of this technological intervention. The results could inform future health interventions and policies aimed at improving medication adherence, ultimately contributing to better health outcomes and reduced healthcare costs in the region.
